# Particulate Size of Microalgal Biomass Affects Hydrolysate Properties and Bioethanol Concentration

**DOI:** 10.1155/2014/435631

**Published:** 2014-05-29

**Authors:** Razif Harun, Michael K. Danquah, Selvakumar Thiruvenkadam

**Affiliations:** ^1^Department of Chemical Engineering, Monash University, Clayton, VIC 3800, Australia; ^2^Department of Chemical and Environmental Engineering, Universiti Putra Malaysia, 43400 Serdang, Malaysia; ^3^Department of Chemical and Petroleum Engineering, Curtin University of Technology, 98009 Sarawak, Malaysia

## Abstract

Effective optimization of microalgae-to-bioethanol process systems hinges on an in-depth characterization of key process parameters relevant to the overall bioprocess engineering. One of the such important variables is the biomass particle size distribution and the effects on saccharification levels and bioethanol titres. This study examined the effects of three different microalgal biomass particle size ranges, 35 **μ**m ≤ *x* ≤ 90 **μ**m, 125 **μ**m ≤ *x* ≤ 180 **μ**m, and 295 **μ**m ≤ *x* ≤ 425 **μ**m, on the degree of enzymatic hydrolysis and bioethanol production. Two scenarios were investigated: single enzyme hydrolysis (cellulase) and double enzyme hydrolysis (cellulase and cellobiase). The glucose yield from biomass in the smallest particle size range (35 **μ**m ≤ *x* ≤ 90 **μ**m) was the highest, 134.73 mg glucose/g algae, while the yield from biomass in the larger particle size range (295 **μ**m ≤ *x* ≤ 425 **μ**m) was 75.45 mg glucose/g algae. A similar trend was observed for bioethanol yield, with the highest yield of 0.47 g EtOH/g glucose obtained from biomass in the smallest particle size range. The results have shown that the microalgal biomass particle size has a significant effect on enzymatic hydrolysis and bioethanol yield.

## 1. Introduction

The utilization of microalgae to produce a variety of products such as fine organic chemicals, food, animal feed, and food supplements have been discovered in the past [1–3]. Current interest has been on the development of biofuels, such as bioethanol, from microalgae as a nonedible feedstock. Aside from its renewable and sustainable benefits, the high carbohydrate composition of microalgal biomass can be converted to fermentable sugars for microbial conversion to bioethanol [4, 5]. One of such biomass saccharification methods is via enzymatic hydrolysis.

Enzymatic hydrolysis is a well-established process and provides mild operating conditions, high sugar yields, high selectivity, and minimal by-products formation [6, 7], hence a more preferred method of hydrolyzing fermentation substrates. However, process conditions and parameters during enzymatic hydrolysis require detailed optimization for maximum product conversion. One of the important parameters that influence the effectiveness of enzymatic hydrolysis is biomass particle size. Fundamentally, smaller particle size biomass presents a large specific surface area, thus increasing the contact areas between the enzymes and the interparticle bonding of the material during the hydrolysis process [8].

Previous attention has been focused on the effect of particle size on enzymatic hydrolysis of either cellulosic (such as cotton, plant, and fibers) or lignocellulosic biomass (such as corn, sugarcane, and wheat). Pedersen and Meyer [9] reported that smaller biomass particle size (53–149 *μ*m) increased glucose release up to 90% after 24 h hydrolysis of wheat straw biomass. The finding was in accordance with those reported by Dasari and Eric Berson [10] and Carvalho et al. [11] who used sawdust and lemon, respectively, as hydrolysis substrates. Biomass particulate size reduction also results in enhancing the hydrolysis rate [10, 12]. This can be explained by the easy access to enzyme active sites by smaller biomass particles. Contrary to this, Ballesteros et al. [13, 14] have reported that larger particle size biomass significantly increases hydrolysis rates and sugar recoveries (particularly glucose) compared to smaller particle size biomass. These conflicting views call for further studies on the characteristic effects of biomass particle size on the degree and effectiveness of enzymatic hydrolysis. To the best of our knowledge, no similar work has been performed on the carbohydrates of microalgae biomass and the concomitant effect on bioethanol yields. Therefore, this study aims to investigate the effect of particle size on enzymatic hydrolysis of microalgal biomass. The glucose yields and the physical properties of the substrate during the hydrolysis process are examined and discussed. Also, the kinetic investigation of enzyme hydrolysis and the effects on glucose and bioethanol yields are presented.

## 2. Materials and Methods

### 2.1. Substrate Preparation

Culture samples of* Chlorococcum infusionum *obtained from Bio-fuels Pty Ltd (Victoria, Australia) were centrifuged (Heraeus Multifuge 3 S-R, Germany) at 4500 × g for 10 mins and the supernatant was discarded. The microalgal cake was dried in a laboratory oven at 60°C for 24 h (Model 400, Memmert, Germany). The dried biomass was pulverized for 1 min using a hammer mill (N.V Tema, Germany). The different particle sizes were separated by passing the milled sample through a series of cascaded stainless steel sieves (until the desired biomass sizes were partitioned in the following ranges: 35 *μ*m ≤ *x* ≤90 *μ*m, 125 *μ*m ≤ *x* ≤ 180 *μ*m, and 295 *μ*m ≤ *x* ≤425 *μ*m). The samples were stored at room temperature before further analysis.

### 2.2. Enzyme Activity

The enzymes used in this study were cellulase from* Trichoderma reesei* (ATCC 26921) and cellobiase from* Aspergillus niger* (Novozyme 188), purchased from Sigma Aldrich, Australia. The activity of cellulase measured at 1.0 units/mg solid means that one unit of cellulase liberates 1.0 *μ*mol of glucose from cellulose in 1 h at pH 5.0. The cellobiase activity was determined as 250 units/mg.

### 2.3. Enzymatic Hydrolysis

Varying quantities of microalgal biomass in powder form (0.2–1.0 g) within three different particle size ranges 35 *μ*m ≤ *x* ≤ 90 *μ*m, 125 *μ*m ≤ *x* ≤ 180 *μ*m, and 295 *μ*m ≤ *x* ≤ 425 *μ*m were loaded with a constant cellulase mass of 20 mg and a cellobiase volume of 1.0 mL. The samples were hydrolysed in shake flasks with 10 mM of 100 mL sodium acetate buffer at pH 4.8 and were placed in an incubator (LH Fermentation Ltd., Buckinghamshire, England) at 40°C for 48 h with 200 RPM agitation. Samples were taken at 5 h intervals and the enzymatic hydrolysis process was halted by heating the hydrolysate to ~90°C for 10 min. The samples were then cooled to room temperature and stored in a freezer at −75°C (Ultraflow freezer, Plymouth, USA) for further analysis.

### 2.4. Bioethanol Production

*Saccharomyces cerevisiae*, purchased from Lalvin, Winequip Products Pty Ltd. (Victoria, Australia), was used in the microbial fermentation process for bioethanol production. The culture was prepared by dissolving 5.0 g of dry yeast powder in 50 mL sterile warm water (~40°C) and the pH was adjusted to 7 by 1 M NaOH addition. The yeast was cultured in YDP medium with composition in g/L given as follows: 10 g yeast extract, 20 g peptone, and 20 g glucose. The yeast was harvested after 24 h and washed to eliminate the sugars then transferred into 500 mL Erlenmeyer flask containing 100 mL of the sugar-containing liquid medium obtained after the hydrolysis process. The flasks were tightly sealed and nitrogen gas was bubbled through to create an oxygen-free environment for bioethanol production. The flasks were incubated at 30°C under 200 RPM shaking. The pH was maintained at 7 by adding 1 M NaOH solution. The fermentation continued for 50 h and samples for analysis were taken after every 4 h.

### 2.5. Chemical Analysis

The biomass was pretreated using a sonicator to break down the cell walls. Phenol-sulphuric acid method was used to quantify the total carbohydrate in the biomass. Note that Table 1 is a presaccharification data, presenting the existence of different carbohydrate forms entrapped in the microalgae system. Microalgal biomass and the hydrolysate compositions were analyzed by HPLC using a 250 mm × 4.6 mm Prevail Carbohydrate ES Column. The HPLC system consists of the following accessory instruments: a detector (ELSD, Alltech 3300), quaternary gradient pump (Model 726, Alltech), degasser (Model 591500M Elite degassing system, Alltech), autosampler (Model 570, Alltech), and system controller (Model 726300M, Alltech). The mobile phase was a mixture of acetonitrile and water (85 : 15) and the operating flow rate was 1 mL/min. 30 *μ*L sample was injected at 50°C. The sample was filtered through a 13 mm membrane filter prior to injection. The sugar concentrations were evaluated using a calibration curve generated from HPLC-grade sugars.

The ethanol concentration was analyzed using gas chromatography (GC) (Model 7890A, Agilent, USA). The GC unit consists of an autosampler, flame ion detector (FID), and HP-FFAP column (50 m ×  0.20 mm × 0.33 *μ*m). The injector, detector, and oven temperatures were maintained at 150°C, 200°C, and 120°C, respectively. Nitrogen gas was used as the carrier gas. The bioethanol concentration was quantified using a calibration curve prepared by injecting different concentrations of a standard ethanol (0.1–10% v/v).

### 2.6. Fourier Transform Infrared Spectroscopy (FTIR)

The polymorphs of the resulting hydrolysate from the hydrolysis process were determined by FTIR. FTIR spectra of hydrolysed samples were recorded on a Nicolet 6700 FTIR (Fischer Scientific, Australia) equipped with Thermo Scientific iD3 ATR accessory (Fischer Scientific, Australia), and the spectra were run and processed with OMNIC software (Version 7.0 ThermoNicolet). The dried hydrolysis samples were loaded on the sample holder and the spectrum was recorded at an average of 32 scans with a spectral resolution of 4 cm^−1^ from 400 to 4000 cm^−1^. Sample spectra were recorded as absorbance values at each data point in triplicates.

### 2.7. Viscosity Measurement

The hydrolysate viscosities were determined using a modular advanced rheometer system (Haake Mars, Thermo Electron Corp., Germany). The system is equipped with a stainless steel measuring plate (MP 660, 60 mm) and a rotor (PP60H, 60 mm). The temperature was set to 30°C, the frequency was maintained at 1.5 Hz, and the gap between the parallel plates was kept at 1 mm. The hydrolysed samples were measured for 5 min at different shear rates ranging from 50 to 500 s^−1^.

## 3. Results and Discussion

### 3.1. Substrate Carbohydrate Composition

According to Table 1, carbohydrate constitutes up to 32% of the dry weight of* C. infusionum* biomass with the major fermentable sugar component being glucose (15.2%), followed by xylose (9.5%), mannose (4.9%), and galactose (2.9%). This strain also contains starch at 11.3% dry weight. The total carbohydrates present in the biomass could be made available for bioethanol production under optimal saccharification and microbial fermentation conditions. The remaining biomass composition could represent lipids, protein, and ash that is available in microalgal strain. Unlike both red and brown algae, the cell wall of most green algae has high cellulose content, ranging up to 70% of the dry weight [15, 16]. The composition of the carbohydrate content in the unicellular microalgal specie per unit mass does not vary greatly among fractions of different particle size. For intact microalgae cells, the carbohydrates are well distributed within the cell membrane and this gives a uniform carbohydrate composition in the membrane.

### 3.2. FTIR Analysis

**Figure 1:**
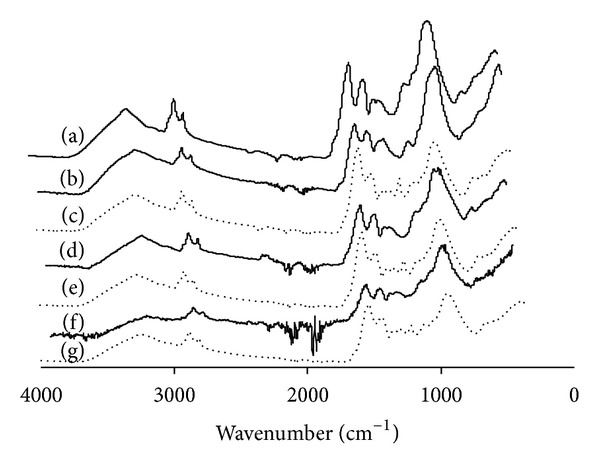
FTIR spectra for microalgal biomass with different particle sizes under different Cases. (a) Nonpretreated microalgal biomass (original powdered sample); Case 1 (cellulase only): (b) 35 *μ*m ≤ *x* ≤ 90 *μ*m, (d) 125 *μ*m ≤ *x* ≤ 180 *μ*m, and (f) 295 *μ*m ≤ *x* ≤ 425 *μ*m; Case 2 (cellulase + cellobiase): (c) 35 *μ*m ≤ *x* ≤ 90 *μ*m, (e) 125 *μ*m ≤ *x* ≤ 180 *μ*m, and (g) 295 *μ*m ≤ *x* ≤ 425 *μ*m.

The spectra of hydrolyzed biomass with different particle sizes were examined using FTIR techniques and the results are shown in Figure 1. Two types of hydrolysates were compared in this study: single enzyme hydrolysate with only cellulase and double enzyme hydrolysate with both cellulase and cellobiase. These two scenarios are denoted by Case 1 and Case 2, respectively. The FTIR spectra represent samples taken at the end of the hydrolysis process. The spectrum of nonpretreated powdered microalgae within the size range of 295 *μ*m ≤ *x* ≤ 425 *μ*m was analyzed for comparison. According to Murdock and Wetzel [17], the reference absorption peaks for major microalgal compositions are ~1100–900 cm^−1^ for polysaccharides (cellulose and starch), ~2970–2850 cm^−1^ for lipids, and 1750–1500 cm^−1^ for proteins and carboxylic groups. Since we wish to convert complex carbohydrates in the biomass to produce fermentable sugars for bioethanol production, only polysaccharide peaks are of interest. The microalgal biomass used in this study showed a relatively high amount of polysaccharides since a strong absorption peak was recorded around 1100 cm^−1^ to 1000 cm^−1^ in the powdered microalgal sample as summarized in Figure 1. It was observed that the degree of polysaccharides absorption decreased as the biomass particle size decreased. This indicates that more polysaccharides were converted to fermentable sugars in the case of biomass with smaller particle size during the hydrolysis process. Based on the individual spectrum, sugar conversions were calculated by referring to the peak heights of nonpretreated samples. The hydrolysis of cellulose with the addition of cellobiase (Case 2) generated hydrolysis conversion of 90, 78, and 64% of the biomass in the particle size ranges 35 *μ*m ≤ *x* ≤ 90 *μ*m, 125 *μ*m ≤ *x* ≤ 180 *μ*m, and 295 *μ*m ≤ *x* ≤ 425 *μ*m, respectively. A lower degree of hydrolysis was observed without cellobiase addition (Case 1) of 41, 29, and 18% for biomass in the particle size ranges 35 *μ*m ≤ *x* ≤ 90 *μ*m, 125 *μ*m ≤ *x* ≤ 180 *μ*m, and 295 *μ*m ≤ *x* ≤ 425 *μ*m, respectively. Cellulase contains cellobiohydrolases, endoglucanases, and *β*-glucosidase that function to efficiently hydrolyse cellulose. The hydrolysis of cellulose to cellobiose is the rate-limiting step, and this limitation is resolved by cellobiohydrolases which hydrolyse cellulose to cellobiose and cellotriose. However, the small amount of *β*-glucosidase in cellulase hinders the cellulolysis process; hence, the addition of *β*-glucosidase helps cellulase to hydrolyse the intermediate product, cellobiose, to form glucose in a faster reaction time while minimizing product inhibition during the cellulolytic process [18–25]. Furthermore, the kinetics of molecular activation drawdown is faster in the double enzyme case and this favors forward production of fermentable subunits during the hydrolysis process. The total crystallinity index (TCI) of the hydrolyzed biomass was calculated as reported by Nelson and O'Connor [19]. From the calculations, the TCI of all the hydrolysed samples decreased when compared with the nonhydrolysed biomass. Decreasing biomass crystallinity has been reported to increase enzymatic hydrolysis rates [26]. Although the polysaccharides were degraded during hydrolysis, FTIR spectra analysis showed that the structure of the hydrolysed monomers remained intact for bioethanol production.

### 3.3. Glucose Yield

Table 2 shows the yield of glucose for different assays. The rate of glucose release was rapid at the beginning of hydrolysis and slowed down until the end of the hydrolysis process. This profile is typical of batch hydrolysis [9]. Note that the enzymes involved in the study are not hydrolysing starch composition thus not accounted for potential glucose for the fermentation process. It was found that biomass with smaller particle size generated higher glucose yields and this observation was the same for both Case 1 and Case 2. The highest glucose yields were 75.45 mg/g biomass and 134.73 mg/g biomass for Cases 1 and 2, respectively, for biomass in the smallest particle size range of 35 *μ*m ≤ *x* ≤ 90 *μ*m. The lowest glucose yields were 26.01 mg/g biomass and 61.55 mg/g biomass for Cases 1 and 2, respectively, for biomass in the largest particle size range of 295 *μ*m ≤ *x* ≤ 425 *μ*m. Smaller biomass particle size increases the interactions with the enzymes during hydrolysis due to the presence of a large exposed surface area [12]. Hence, smaller microalgal biomass particle size is required to achieve higher glucose yield. The amount of microalgal biomass loaded in the hydrolysis process also showed a significant effect on glucose yields. Although the same microalgal biomass particle size was used in assay numbers 1, 2, and 3, different glucose yields of 111.08 mg/g biomass, 125.77 mg/g biomass, and 134.73 mg/g biomass were achieved. When examining the effect of different substrate concentrations on glucose yield within the same particle size range in Case 2, it was found that the glucose yield increased with increasing substrate concentration. This trend was however not observed in Case 1 containing cellulose enzyme. Therefore, high yield of glucose from increasing substrate concentration is dependent on the balanced composition of cellulosic enzyme components to minimize product inhibition [27]. Furthermore, a significant increase in glucose yield was observed when the second enzyme (cellobiase) was introduced to the assays (Case 2). The glucose yields in Case 2 were almost the double compared to those obtained in Case 1. From the collision theory perspective, the kinetics of molecular activation drawdown is faster in the double enzyme case and this favors forward production of fermentable subunits. The kinetics of this double enzyme effect is demonstrated with the scheme below.

Mechanism of enzymatic hydrolysis of cellulase, Case 1:

(1) $$\begin{array}{c} S+E\overset{k_{1}}{\underset{k_{-1}}{⟺}}\text{SE}\overset{k_{2}}{⟶}P+E, \end{array}$$

where *S* is the substrate concentration, *E* is the enzyme concentration, SE is the concentration of substrate-enzyme complex, *P* is the product concentration, and *k*_1_, *k*_−1_, *k*_2_ are rate constants.

The rates of change in SE concentration and product formation are

(2) dSEdt=k1S×E−k−1SE−k2SE,

(3) $$\begin{array}{c} \frac{dP}{dt}=k_{2}\text{SE}. \end{array}$$

For substrate mass balance, the substrate concentration (*S*) is written as

(4) $$\begin{array}{c} S=S_{o}-\text{SE}-P. \end{array}$$

Substituting (4) into (2) gives

(5) $$\begin{array}{c} \frac{d\text{SE}}{dt}=k_{1}\left(S_{0}-\text{SE}-P\right)\times E-k_{-1}\text{SE}-k_{2}\text{SE}. \end{array}$$

Applying equilibrium and steady state conditions to (5) gives

(6) $$\begin{array}{c} \text{SE}=\frac{\left(S_{0}-P\right)E}{K_{e}+E}, \end{array}$$

where *K*_*e*_ is the equilibrium constant

(7) $$\begin{array}{c} K_{e}=\frac{k_{-1}+k_{2}}{k_{1}}. \end{array}$$

The simplified equation (6) may be written as follows at initial conditions:

(8) $$\begin{array}{c} {\left(\frac{dP}{dt}\right)}_{P_{0}}=\frac{k_{2}\;S_{0}\;E_{0}}{K_{e}+E_{0}}, \end{array}$$

where (*dP*/*dt*)_*P*_0__ denotes the product formation at the initial conditions.

The mechanism of the enzymatic hydrolysis of cellulase (*E*_1_) and cellobiase (*E*_2_), Case 2, is

(9) $$\begin{array}{c} S_{C}+E_{1}\overset{k_{1}}{\underset{k_{-1}}{⟺}}S_{C}E_{1}\overset{k_{2}}{⟶}S_{\text{CB}}+E_{1},\\ S_{\text{CB}}+E_{2}\overset{k_{1}^{\prime }}{\underset{k_{-1}^{\prime }}{⟺}}S_{\text{CB}}E_{2}\overset{k_{2}^{\prime }}{⟶}P+E_{2}, \end{array}$$

where  *S*_*C*_,  *S*_CB_ , and  *P*  are concentrations of cellulose, cellobiose, and glucose, respectively.

By following the same procedure as in Case 1, simplified equation for Case 2 at the initial conditions is

(10) $$\begin{array}{c} {\left(\frac{dP}{dt}\right)}_{P_{0}}=\frac{k_{2}^{\prime }\;E_{2}\left(S_{C_{0}}-S_{C}E_{1_{0}}-S_{CB_{0}}\right)}{K_{e}^{\prime }+E_{2_{0}}}, \end{array}$$

(11) $$\begin{array}{c} K_{e}^{\prime }=\frac{k_{2}^{\prime }+k_{-1}^{\prime }}{k_{1}^{\prime }}, \end{array}$$

where *k*_1_′, *k*_−1_′, and *k*_2_′ are rate constants and *K*_*e*_′ is the equilibrium constant.

Equations (8) and (10) can be rewritten as follows.

For Case 1,

(12) $$\begin{array}{c} \frac{1}{v}=\frac{K_{e}}{V_{0}}\left[\frac{1}{S_{o}}\right]+\frac{1}{V_{0}}, \end{array}$$

where

(13) $$\begin{array}{c} V_{0}=k_{2}E. \end{array}$$

For Case 2,

(14) $$\begin{array}{c} \frac{1}{v}=\frac{K_{e}^{\prime }}{V_{0}^{\prime }}\left[\frac{1}{S_{\text{CB}}}\right]+\frac{1}{V_{0}^{\prime }}, \end{array}$$

where

(15) $$\begin{array}{c} V_{0}^{\prime }=k_{2}^{\prime }\left(E_{T}-S_{C}E_{1}\right). \end{array}$$

From the mathematical derivation, *K*_*m*_ which is the equilibrium constant is *K*_*e*_ for Case 1 and *K*_*e*_′ for Case 2. Also, *V*_max⁡_ which is the maximum forward velocity is 1/*V*_0_ for Case 1 and 1/*V*_0_′ for Case 2, where *V*_0_ and *V*_0_′ occur at their respective initial enzyme concentration. As can be seen in Table 3, Lineweaver-Burk plot analysis of (12) and (14) shows that the *K*_*m*_ value for Case 1 is higher than Case 2 and the *V*_max⁡_ value for Case 1 is lower than Case 2. The lower value of *K*_*m*_ and the higher value of *V*_max⁡_ obtained from Case 2 confirm that the introduction of cellobiase significantly increases the combined enzyme-substrate affinity and the hydrolysis rate.

### 3.4. Ethanol Yield

**Figure 2:**
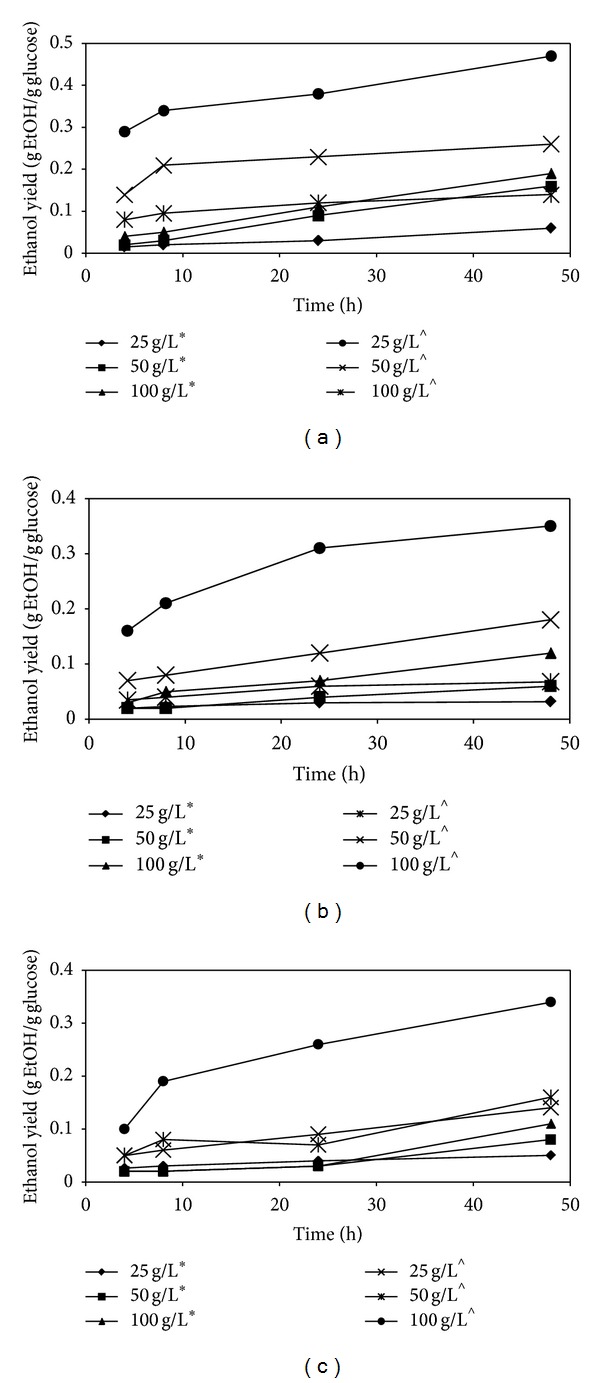
Yield of bioethanol after 48 h fermentation of microalgal biomass with different particle sizes for both cases: (a) 35 *μ*m ≤ *x* ≤ 90 *μ*m, (b) 125 *μ*m ≤ *x* ≤ 180 *μ*m, and (c) 295 *μ*m ≤ *x* ≤ 425 *μ*m (*Case 1: cellulase; ^∧^Case  2: cellulase + cellobiase).

The produced hydrolysates were used as substrates in* Saccharomyces cerevisiae* fermentations for bioethanol production. This yeast strain has widely been utilized for bioethanol production because it is easy to culture and has a high ethanol tolerance. This could allow fermentation to continue under 16-17% v/v ethanol concentrations [28].  Figure 2 shows the bioethanol yields for both Cases 1 and 2 using biomass with different particle sizes. The trend in bioethanol yield for the different particle size biomass was in agreement with the glucose yields; biomass with smaller particle size displayed higher glucose concentrations to generate higher bioethanol yields. It can be observed that available glucose in the hydrolysate was completely consumed after 48 h of fermentation. The highest bioethanol yield of 0.47 g ethanol/g glucose was obtained when hydrolysed under Case 2 with the smallest particle size biomass (35 *μ*m ≤ *x* ≤ 90 *μ*m) at 100 g/L microalgae concentration, whereas the lowest bioethanol yield of 0.05 g ethanol/g glucose was obtained when hydrolysed under Case 1 with the largest particle size biomass (295 *μ*m ≤ *x* ≤ 425 *μ*m) at 25 g/L microalgae concentration. Assays in Case 2 produced up to 50% more bioethanol yields than the assays in Case 1, reaching a maximum bioethanol yield of 0.47 g/g glucose compared to 0.19 g/g glucose, as represented by assay number 3 in both cases. Hydrolysate produced in the presence of cellobiase generated higher bioethanol yields due to the presence of high glucose concentrations.

### 3.5. Viscosity Analysis

**Figure 3:**
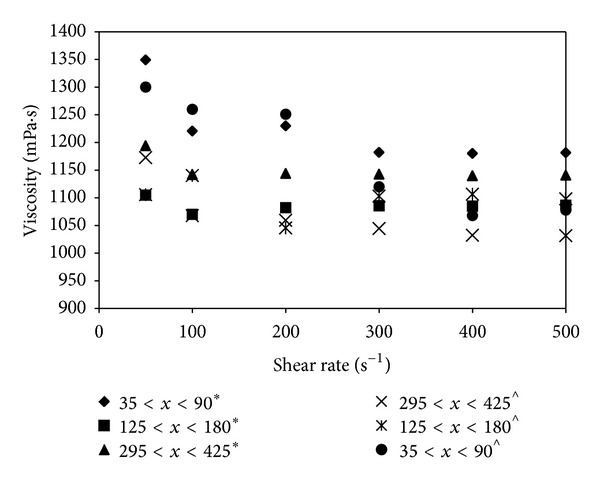
Viscosity versus shear rate for the different particle size biomass. A decreasing trend in viscosity was observed with increasing shear rate (*Case 1: cellulase; ^∧^Case  2:cellulase + cellobiase; substrate concentration: 100 g/L).

The purpose of the viscosity study is to understand the influence of the rheological properties of the hydrolysate during hydrolysis and how this affects the fermentation process for bioethanol production. The viscosity measurements were performed under different shear rates (50–500 s^−1^) using hydrolysates obtained from biomass with different particle sizes for an equivalent substrate concentration of 100 g/L.  Figure 3 shows the viscosity data of the different particle size biomass for both Cases 1 and 2 with samples taken after the hydrolysis process. A decreasing trend of viscosity was observed with increasing shear rate and biomass with smaller particle sizes displaying higher viscosities. For biomass in the same particle size range, Case 2 showed a slightly higher viscosity than Case 1. The effective enzyme-substrate interactions associated with smaller particle size biomass result in a more viscous hydrolysate than large size particles as more water molecules are consumed per unit volume, exceeding the reduction of total solids concentration [29].

**Figure 4:**
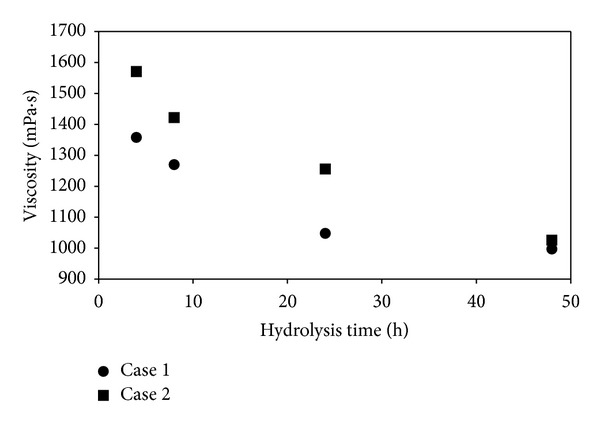
Hydrolysate viscosity profiles during hydrolysis for single-enzyme (Case 1: cellulase) and double-enzyme (Case 2: cellulase + cellobiase) conditions. The viscosity of the hydrolysates in both cases decreased with hydrolysis time. The data presented is for assay number 1 in both cases (substrate concentration: 25 g/L).

We also studied the viscosity profile of the hydrolysates over the time course of hydrolysis and the data is presented in Figure 4. The hydrolysate viscosities for both Cases 1 and 2 reduced with hydrolysis time with a significant decrease which was observed at the initial stage of hydrolysis between 4 and 24 h. This is probably due to the faster initial kinetics, structural changes, and/or the release of intercalating molecules in the cell wall. Decreasing viscosity during hydrolysis is caused by cellulose degradation as the structure and solid concentration change during the cellulolytic activity caused by the enzymes [10].

**Figure 5:**
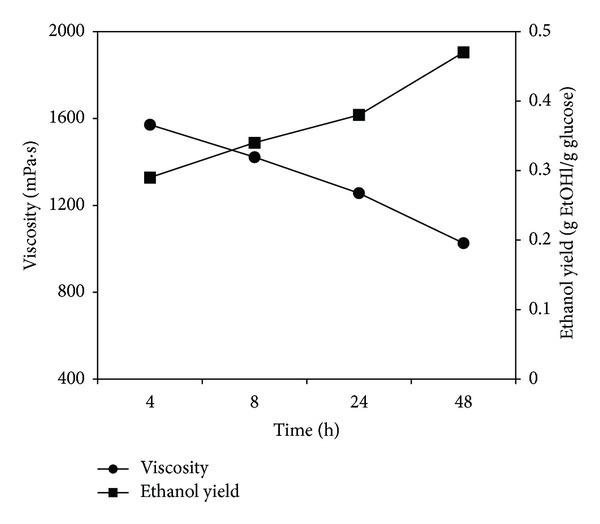
Relationship between hydrolysate viscosity and bioethanol yield. Bioethanol yield increases with lower hydrolysate viscosities. The data presented is for assay number 1 of Case 2 (substrate concentration: 25 g/L).

The profiles of viscosity and bioethanol production were superimposed to understand their relationship during the enzymatic hydrolysis process as shown in Figure 5. It can be seen that bioethanol yield increases with lower viscosities. This trend also matches glucose yields as higher released glucose produces higher bioethanol yields. The results show that less viscous slurry is required to produce high glucose yields under effective mixing.

## 4. Conclusion

This paper is the premier study on the effect of particle size of microalgal biomass on enzymatic hydrolysis and bioethanol production. The results show that the highest glucose and bioethanol yields were obtained using biomass with smaller particle size (35 *μ*m ≤ *x* ≤ 90 *μ*m) at a substrate concentration of 100 g/L. The addition of the second enzyme, cellobiase, increases the glucose yield, thus increasing the bioethanol yield. This was confirmed by a kinetic investigation of the double enzyme process using the rapid equilibrium model. The viscosity of the hydrolysate also influences glucose yield. Lower viscosities result in higher glucose yields. Overall, microalgal biomass particle size has a significant effect on enzymatic hydrolysis and bioethanol production.

## Figures and Tables

**Table 1:** Biomass composition of the microalgal species.

Component	Composition (% w/w)
Total carbohydrate	32.52
Xylose	9.54
Mannose	4.87
Glucose	15.22
Galactose	2.89
Starch	11.32
Others*	56.16

*Lipids, protein, and ash.

**Table 2:** Yield of glucose released after 48 h of hydrolysis.

Assay number	Particle size, µm	Algae loading, g/L	mg glucose/g algal biomass	
			Cellulase (Case 1)	Cellulase + cellobiase (Case 2)
1	35 ≤ *x* ≤ 90	25	54.21	111.08
2	35 ≤ *x* ≤ 90	50	44.48	125.77
3	35 ≤ *x* ≤ 90	100	75.45	134.73
4	125 ≤ *x* ≤ 180	25	30.24	68.79
5	125 ≤ *x* ≤ 180	50	27.96	85.88
6	125 ≤ *x* ≤ 180	100	27.63	114.54
7	295 ≤ *x* ≤ 425	25	26.01	68.79
8	295 ≤ *x* ≤ 425	50	26.43	92.61
9	295 ≤ *x* ≤ 425	100	30.24	102.29

**Table 3:** *K*_*m*_ and *V*_max⁡_ for hydrolysis of cellulose by cellulase (Case 1) and cellulase + cellobiase (Case 2).

Case number	Enzyme	Hydrolysis of cellulose	
		*K*_*m*_	*V*_max⁡_
1	Cellulase	18.81	35.05
2	Cellulase + cellobiase	18.23	135.83

## Abbreviations

*S*: Substrate concentration, g/L
*E*: Enzyme concentration, g/L
SE: Concentration of substrate-enzyme complex
*P*: Product concentration, g/L
*k*_1_, *k*_−1_, *k*_2_, *k*_1_′, *k*_−1_′, and *k*_2_′: Rate constants, g/L
*K*_*e*_, *K*_*e*_′: Equilibrium constant, g/L
(*dP*/*dt*)_*P*_0__: Product formation at the initial conditions
*E*_1_: Enzyme I (cellulase) concentration, g/L
*E*_2_: Enzyme II (cellobiase) concentration, g/L
*S*_*C*_: Cellulose concentration, g/L
*S*_CB_: Cellobiose concentration, g/L
*S*_*C*_*E*_1_: Concentration of cellulose-cellulase complex
*S*_CB_*E*_2_: Concentration of cellobiose-cellobiase complex
*K*_*m*_: Michaelis constant, g/L
*V*_max⁡_: Maximum rate of hydrolysis, g/L·min.
